# Supplementary material for: Two Novel Genera, Neostemphylium and Scleromyces (Pleosporaceae) from Freshwater Sediments and Their Global Biogeography
Source: J Fungi (Basel). 2022 Aug 17;8(8):868. doi: 10.3390/jof8080868 (PMC9409710; doi:10.3390/jof8080868)
Supplement: Supplementary file 1 [file jof-08-00868-s001.zip › jof-1847094-supplementary.pdf]

Journal of
Fungi

MDPI

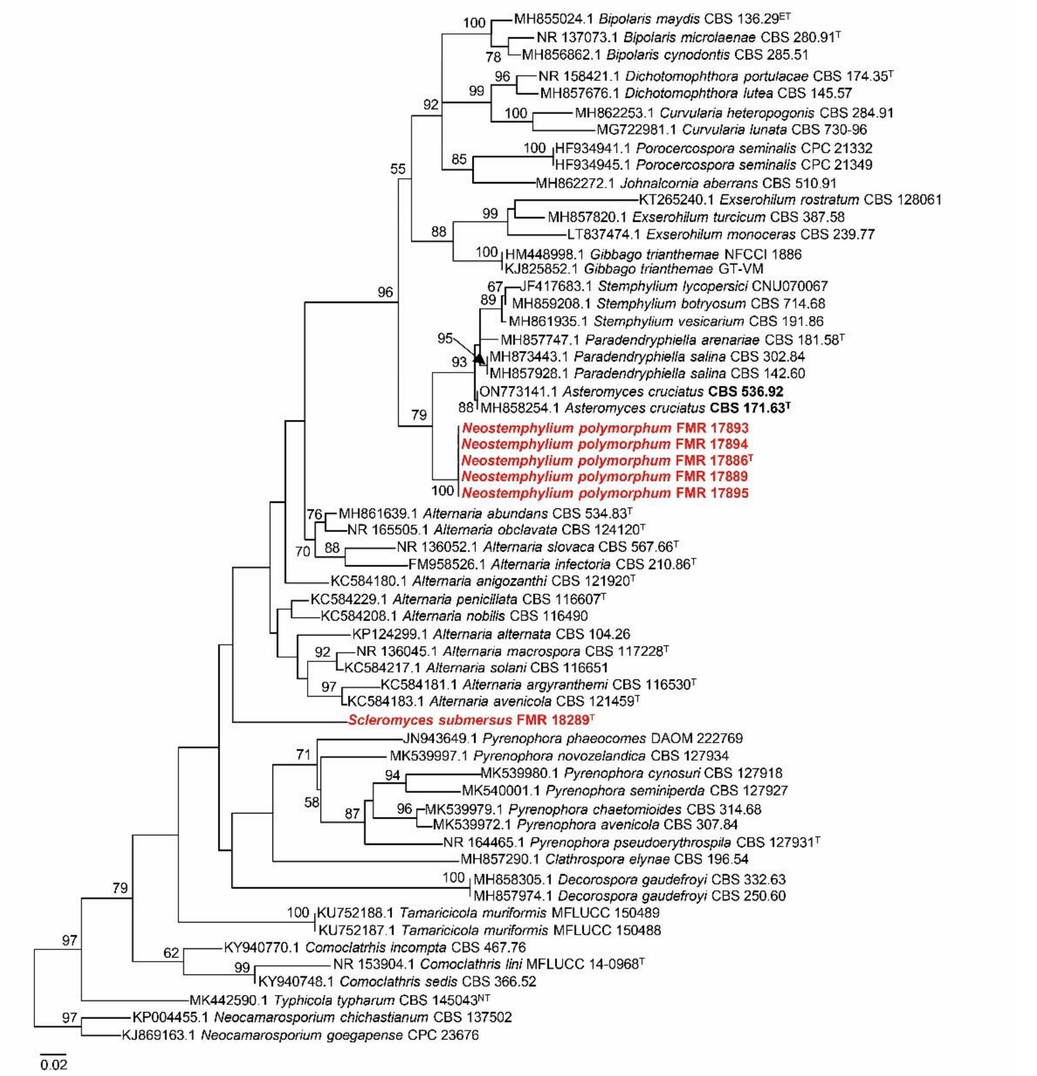

Figure S1. RAxML tree of the *Pleosporaceae* family from ITS including the strains recovered from freshwater sediments studied in this work. The strains studied in this work are in bold and in red. Determined by MEGA software v.6, the best nucleotide substitution model for ML analysis was K2+G+I. The aligned data set was 531 bp long, with 217 variable sites and 198 phylogenetically informative. Branch lengths are proportional to phylogenetic distance. Bootstrap support values above 50% are indicated on the nodes. The tree is rooted to *Neocamarosporium chichastianum* CBS 137502 and *Neocamarosporium goegapense* CPC 23676. ^{T}= Ex-type strain.

Table S1. Environmental and biogeographical information contained in all ITS1/ITS2 sequences downloaded from the GlobalFungi database included in our analysis (see Figure 2).

Hypothetical Taxa	Sequence	ITS[1]	Sample ID[2]	Primers	Longitude	Latitude	Sample Type	ITS Observed[3]	ITS Total[4]	Freq. R[5]	Biome	MAT[6]	MAP[7]	pH	Geographical Origin	Dominant Plants
Alternaria avenicola	398ac0e08465fe47f2af7374ad912c26	ITS1	2454	ITS1ngs/ITS4ngs	22.1828	58.1061	Soil	1	2643	0.0378	Grassland	7.1	566	NA	Estonia	
Alternaria avenicola	40197eb26ec13e85f78292bafb0faa7f	ITS1	12856	ITS1ngs/ITS4ngs	21.972	58.451	Soil	1	2968	0.0337	Grassland	7.0	591	NA	Estonia	*Platanthera chlorantha*
Alternaria avenicola	b728a7b942d7518ea9b3b8b82cda9821	ITS1	8244	ITS1F/ITS4	-4.25	42.81	Root	4	38391	0.0104	Grassland	10.2	NA	6.61	Spain	*Microthlaspi perfoliatum*
Alternaria avenicola	d52110c7fa2698f97ce9c594036a7a59	ITS1	8244	ITS1F/ITS4	-4.25	42.81	Root	2	38391	0.0052	Grassland	10.2	NA	6.61	Spain	*Microthlaspi perfoliatum*
Alternaria avenicola	d5e3b017e0f32f46e5f2778179f4fb6e	ITS1	252	ITS1F/ITS4	110.527	-66.282	Soil	2	209763	0.0010	Desert	NA	NA	5.91	Antarctica	
Neostemphylium polymorphum	005722a617645f9fee72b3bef5fb6873	ITS1	4773	ITS1F/ITS4	147.614	-42.794	Soil	2	156717	0.01	Grassland	10.9	615	5.6	Australia	
Neostemphylium polymorphum	0a38b3f7b73520b84914b2928c26a46e	ITS1	4773	ITS1F/ITS4	147.614	-42.794	Soil	1	156717	0.006	Grassland	10.9	615	5.5	Australia	
Neostemphylium polymorphum	2d0b551038667839eb5e0ba20c0b20ef	ITS1	4773	ITS1F/ITS4	147.614	-42.794	Soil	2	156717	0.012	Grassland	10.9	615	5.5	Australia	
Neostemphylium polymorphum	32738b33d0714aac20ab9ab19f3071d8	ITS1	4773	ITS1F/ITS4	147.614	-42.794	Soil	3	156717	0.019	Grassland	10.9	615	5.5	Australia	
Neostemphylium polymorphum	3dfb7d5b5c53b75fcc9bc8ac595aa225	ITS1	4773	ITS1F/ITS4	147.614	-42.794	Soil	2	156717	0.012	Grassland	10.9	615	5.5	Australia	
Neostemphylium polymorphum	432c6ddc5a573f2d5bb0adc44a79a377	ITS1	4773	ITS1F/ITS4	147.614	-42.794	Soil	1	156717	0.003	Grassland	10.9	615	5.5	Australia	
Neostemphylium polymorphum	4dd9ffac7a16f1444a2e543471fc3006	ITS1	4773	ITS1F/ITS4	147.614	-42.794	Soil	164	156717	0.104	Grassland	10.9	615	5.5	Australia	
Neostemphylium polymorphum	5666fb2cc1fa5ebed97dfce7d0175259	ITS1	1638	ITS1F/ITS4	147.614	-42.794	Soil	28	122142	0.229	Grassland	10.9	615	5.6	Australia	
Neostemphylium polymorphum	567b99d492e0a9cf157762b3520b2012	ITS1	6768	ITS1F/ITS4	-117.179	46.785	Soil	3	7889	0.380	Cropland	9.3	536	5.6	USA	*Triticum aestivum*
Neostemphylium polymorphum	5dd8920f75870263f9575b604d2ae8a3	ITS1	8233	ITS3_KYO2/ITS4	-2.77389	42.541	Rhizosphere soil	4	89998	0.044	Cropland	13	626	8.2	Spain	*Vitis vinifera*
Neostemphylium polymorphum	7a89eddabaa774d3ea09513e72b7acf9	ITS1	1638	ITS1F/ITS4	147.614	-42.794	Soil	1	122142	0.0081	Grassland	10.9	615	5.6	Australia	
Neostemphylium polymorphum	98148edf126d072bd6fdebb00e2ad6eb	ITS1	4773	ITS1F/ITS4	147.614	-42.794	Soil	2	156717	0.012	Grassland	10.9	615	5.5	Australia	
Neostemphylium polymorphum	a7682143810c8e8673cda2abf1d2f9dc	ITS1	4773	ITS1F/ITS4	147.614	-42.794	Soil	121	156717	0.77	Grassland	10.9	615	5.5	Australia	

Neostemphylium polymorphum	b772018cda8df28137a048d4e2c49c5f	ITS1	1638	ITS1F/ITS4	147.614	-42.794	Soil	1	122142	0.0081	Grassland	10.9	615	5.6	Australia	
Neostemphylium polymorphum	bf84e5d488b9b365d4d3565cd263726b	ITS1	4773	ITS1F/ITS4	147.614	-42.794	Soil	4	156717	0.025	Grassland	10.9	615	5.5	Australia	
Neostemphylium polymorphum	c09ecbc23cf5699e459aa7592c3bf50f	ITS1	4773	ITS1F/ITS4	147.614	-42.794	Soil	2	156717	0.012	Grassland	10.9	615	5.5	Australia	
Neostemphylium polymorphum	c3188249cad06c49b99df58c645b4192	ITS1	1638	ITS1F/ITS4	147.614	-42.794	Soil	1	122142	0.008	Grassland	10.9	615	5.6	Australia	
Neostemphylium polymorphum	c428603cddb061ed5d5f0eba7b64d561	ITS1	1638	ITS1F/ITS4	147.614	-42.794	Soil	1	122142	0.008	Grassland	10.9	615	5.6	Australia	
Neostemphylium polymorphum	ccb6952f6a039cb0d798c3393fcdfdff	ITS1	16091	ITS1F/ITS2	-123.000	43.93	Root	48	48773	0.984	Wetland	12.4	1029	5.9	USA	*Camassia quamash*
Neostemphylium polymorphum	d3d82840d91aaa96410995dd98137cce	ITS1	4773	ITS1F/ITS4	147.614	-42.794	Soil	2	156717	0.012	Grassland	10.9	615	5.5	Australia	
Neostemphylium polymorphum	d6138ce3588d37ae8e12eaee65f2c3ae	ITS1	4773	ITS1F/ITS4	147.614	-42.794	Soil	2	156717	0.012	Grassland	10.9	615	5.5	Australia	
Neostemphylium polymorphum	dbeeb6cf718c5ee470f0266ec74203b2	ITS1	4773	ITS1F/ITS4	147.614	-42.794	Soil	2	156717	0.012	Grassland	10.9	615	5.5	Australia	
Neostemphylium polymorphum	ede3c861bbc52b33af809182642dc525	ITS1	4773	ITS1F/ITS4	147.614	-42.794	Soil	3	156717	0.019	Grassland	10.9	615	5.5	Australia	
Neostemphylium polymorphum	f0cf55b2f85219853e589577d8edacef	ITS1	1638	ITS1F/ITS4	147.614	-42.794	Soil	1	122142	0.008	Grassland	10.9	615	5.6	Australia	
Neostemphylium polymorphum	f935ebc9a26ad00f9d988404c41a2b0e	ITS1	4773	ITS1F/ITS4	147.614	-42.794	Soil	5	156717	0.031	Grassland	10.9	615	5.5	Australia	
Neostemphylium polymorphum	fa46ae6fd4793533dfd716cfefd5ff48	ITS1	1638	ITS1F/ITS4	147.614	-42.794	Soil	1	122142	0.008	Grassland	10.9	615	5.6	Australia	
Neostemphylium polymorphum	fa47fed73cc46aef4f127e173148607d	ITS1	4773	ITS1F/ITS4	147.614	-42.794	Soil	121	156717	0.772	Grassland	10.9	615	5.5	Australia	
Neostemphylium sp. ITS1-ENV1	dd1c7e5445d507dbcc01222aa8fb97a9	ITS1	12087	ITS1F/ITS2	-157.663	21.316	Shoot	1	21408	0.046	Forest	24.2	941	NA	USA. Hawai	*Ipomoea sp.*
Neostemphylium sp. ITS1-ENV1	82ef96468f71d5ba4d5eede1a0cc0308	ITS1	1	ITS1F/ITS4	138.659	-35.085	Soil	1	176544	0.005	Woodland	14.2	863	5.7	Australia	
Neostemphylium sp. ITS1-ENV1	c016dc388bf8edbd076a92b0bc4b568c	ITS1	2446	ITS1F/ITS4	138.659	-35.085	Soil	1	315470	0.003	Woodland	14.2	863	5.7	Australia	
Neostemphylium sp. ITS1-ENV1	14f2ed49b592cb3073b8143d503ac537	ITS1	2446	ITS1F/ITS4	138.659	-35.085	Soil	1	315470	0.003	Woodland	14.2	863	5.7	Australia	
Paradendryphiella salina	687b9e04de610c10568b7b873ad7115e	ITS1	16993	ITS1F/ITS2	-155.931	19.6152	Shoot	10	4924	2.030	Forest	20.6	777	NA	USA. Hawai	*Nestegis sandwicensis*
Paradendryphiella salina	84677b6a207aa08c9332a37d2f9f8933	ITS1	3527	ITS1F/ITS2	-155.282	19.6817	Shoot	205	20109	10.19	Forest	17.4	3085	NA	USA. Hawai	*Metrosideros polymorpha*

Paradendryphiella salina	dbf67b714f61957681c87be08c228fa3	ITS1	3527	ITS1F/ITS2	-155.282	19.6817	Shoot	205	20109	10.19	Forest	17.4	3085	NA	USA. Hawai	*Metrosideros polymorpha*
Paradendryphiella salina	ee1d75cd640f2f5799fc8f6c54b02c3c	ITS1	15717	ITS1F/ITS2	-155.268	19.6875	Shoot	67	17385	3.853	Forest	17.7	3126	NA	USA. Hawai	*Vaccinium reticulatum*
Pyrenophora seminiperda	8f68ba966709670a93d593d3764055a6	ITS1	2585	ITS1F/ITS4	138.685	-34.754	Soil	4	72627	0.0055	Woodland	15.9	573	8.3	Australia	
Pyrenophora seminiperda	1feb4971d0a494ae8b100fd166b857ff	ITS1	9312	ITS1F/ITS4	137.957	-32.303	Soil	2	58549	0.0034	Shrubland	NA	NA	8	Australia	
Pyrenophora seminiperda	6b699e4e90e7fa151cbefb5678c486ed	ITS1	2585	ITS1F/ITS4	138.685	-34.754	Soil	2	72627	0.0028	Woodland	NA	NA	8.3	Australia	
Pyrenophora seminiperda	6d9ce6c910a8a3a085c326cc316b792e	ITS1	7850	ITS1F/ITS4	138.707	-34.657	Soil	1	129986	0.0008	Woodland	NA	NA	7.5	Australia	
Pyrenophora seminiperda	74d3099f9f1da65c82b1903ee27330bd	ITS1	13136	ITS1F/ITS2	143.144	-37.315	Soil	1	79861	0.0013	Cropland	NA	587.9	7.2	Australia	*Vitis vinifera*
Pyrenophora seminiperda	7d51963aab8a458308c09713f44d1708	ITS1	3553	ITS1F/ITS4	138.782	-34.617	Soil	2	98422	0.0020	Woodland	NA	NA	7.1	Australia	
Pyrenophora seminiperda	8bed6aed0ab80ead7f7f79d65df7e906	ITS1	3553	ITS1F/ITS4	138.782	-34.617	Soil	2	98422	0.0020	Woodland	16	504	7.1	Australia	
Pyrenophora seminiperda	94409695961e692a0465c3eb30bd38a4	ITS1	16120	ITS1F/ITS2	-105.644	40.0516	Soil	1	4537	0.0220	Tundra	-3.7	757	4.16	USA	
Pyrenophora seminiperda	c8daee186ebb8efab776b0cc985498be	ITS1	3553	ITS1F/ITS4	138.782	-34.617	Soil	2	98422	0.0020	Woodland	16	504	7.1	Australia	
Pyrenophora seminiperda	fef60f5ac4431e0b052ad57134616dd3	ITS1	175	ITS1F/ITS4	138.783	-34.620	Soil	2	41197	0.0049	Woodland	16	504	7.2	Australia	
Scleromyces sp. ITS1-ENV2	f4eda6a824d78112dfabe6c7baccb029	ITS1	3972	ITS1F/ITS4	138.163	-35.655	Soil	2	155747	0.01284	Woodland	15	681	5.8	Australia	
Scleromyces sp. ITS1-ENV3	266ec1268ce5b36dd29748b49f3934f2	ITS1	2065	ITS1F/ITS2	115.107	-30.258	Soil	4	75780	0.0527	Shrubland	19.9	581	7.03	Australia	
Scleromyces sp. ITS1-ENV3	ea68c692f58283a97a7bbfbd702a3438	ITS1	2532	ITS1F/ITS2	115.074	-30.191	Soil	2	64017	0.031	Shrubland	20.2	557	6.55	Australia	
Stemphylium lycopersici	066a94da068657a4a2af4e0e77e0fcfa	ITS1	13126	ITS1F/ITS2	-155.251	19.4116	Shoot	1	14991	0.066	Forest	17.9	2422	NA	USA. Hawai	*Myrsine umbellata*
Stemphylium lycopersici	0fb500d068e8c60dd99fd13d297116fd	ITS1	17317	ITS1F/ITS2	-158.207	21.5814	Shoot	3275	8316	285.594	Forest	24.6	1193	NA	USA. Hawai	*Ipomoea pes-caprae subsp. brasiliensis*
Stemphylium lycopersici	18e228398da1c805a828ecfc18afb583	ITS1	26289	ITS1F/ITS2	-158.207	21.5814	Shoot	9131	14681	621.96	Forest	24.6	1193	NA	USA. Hawai	*Scaevola taccada*
Stemphylium lycopersici	2e33db056dc147d747e7903fdb1fce28	ITS1	30302	ITS1F/ITS2	-159.411	21.8915	Shoot	837	2561	326.825	Forest	24.5	1795	NA	USA. Hawai	*Ipomoea pes-caprae*
Stemphylium lycopersici	4835e53f51c058cda84f62ebb26777a0	ITS1	12667	ITS1F/ITS2	-157.662	21.2919	Shoot	7200	11139	646.376	Forest	24.6	869	NA	USA. Hawai	*Sesbania tomentosa*

Stemphylium lycopersici	6979bd07182d91630c3c7f39cb75c041	ITS1	30302	ITS1F/ITS2	-159.411	21.8915	Shoot	837	2561	326.825	Forest	24.5	1795	NA	USA. Hawai	*Ipomoea pes-caprae*
Stemphylium lycopersici	71d0b2994504b79b69b6809569d8d7c1	ITS1	13710	ITS1F/ITS2	-157.662	21.2015	Shoot	14582	24908	585.434	Forest	24.6	720	NA	USA. Hawai	*Scaevola taccada*
Stemphylium lycopersici	724ed29bd2026dff776c8b266ab099cd	ITS1	26289	ITS1F/ITS2	-158.207	21.5814	Shoot	9131	14681	621.960	Forest	24.6	1193	NA	USA. Hawai	*Scaevola taccada*
Stemphylium lycopersici	7a9cd6851703a9ddecc9cac9130b43bb	ITS1	26289	ITS1F/ITS2	-158.207	21.5814	Shoot	9131	14681	621.960	Forest	24.6	1193	NA	USA. Hawai	*Scaevola taccada*
Stemphylium lycopersici	7c67cdcc5afaa190ba0fd2190ddc8652	ITS1	12667	ITS1F/ITS2	-157.662	21.2919	Shoot	7200	11139	646.376	Forest	24.6	869	NA	USA. Hawai	*Sesbania tomentosa*
Stemphylium lycopersici	7e380924d83cda6f6bd999af61298853	ITS1	226289	ITS1F/ITS2	-158.207	21.5814	Shoot	9131	14681	621.960	Forest	24.6	1193	NA	USA. Hawai	*Scaevola taccada*
Stemphylium lycopersici	8e65316434ecabf8729a020903048972	ITS1	30302	ITS1F/ITS2	-159.411	21.8915	Shoot	837	2561	326.825	Forest	24.5	1795	NA	USA. Hawai	*Ipomoea pes-caprae*
Stemphylium lycopersici	9f431ed2736bb036a8f4d4c53c406b19	ITS1	12667	ITS1F/ITS2	-157.662	21.2919	Shoot	7200	11139	646.376	Forest	24.6	869	NA	USA. Hawai	*Sesbania tomentosa*
Stemphylium lycopersici	af6e7a0137959b378f0b6caf4f9c7b1a	ITS1	12667	ITS1F/ITS2	-157.662	21.2919	Shoot	7200	11139	646.376	Forest	24.6	869	NA	USA. Hawai	*Sesbania tomentosa*
Stemphylium lycopersici	b5b6c72394fe1415c315d7a093388391	ITS1	12087	ITS1F/ITS2	-157.663	21.3160	Shoot	9784	21408	457.025	Forest	24.2	941	NA	USA. Hawai	*Ipomoea sp.*
Stemphylium lycopersici	bc6eaff9b9fbe9af61a187a2da2922b4	ITS1	30302	ITS1F/ITS2	-159.411	21.8915	Shoot	837	2561	326.825	Forest	24.5	1795	NA	USA. Hawai	*Ipomoea pes-caprae*
Stemphylium lycopersici	bfbfb9cc220edab7f7f7461d7a823023	ITS1	12667	ITS1F/ITS2	-157.662	21.2919	Shoot	7200	11139	646.376	Forest	24.6	869	NA	USA. Hawai	*Sesbania tomentosa*
Stemphylium vesicarium	492ed98af71918dd28dc9730203e8dcf	ITS1	492	ITS1F/ITS2	126.955	37.4653	Dust	12	87392	0.13	Anthropogenic	12	1383	NA	South Korea	
Stemphylium vesicarium	84677b6a207aa08c9332a37d2f9f8933	ITS1	4352	ITS1F/ITS2	-155.282	19.6817	Shoot	205	20109	10.19	Forest	17.4	3085	NA	USA. Hawai	*Metrosideros polymorpha*
Stemphylium vesicarium	dbf67b714f61957681c87be08c228fa3	ITS1	4352	ITS1F/ITS2	-155.282	19.6817	Shoot	205	20109	10.19	Forest	17.4	3085	NA	USA. Hawai	*Metrosideros polymorpha*
Stemphylium vesicarium	ed3318e54295c1c4c2ae7e737c52aa60	ITS1	1350	ITS1F_KYO1/ITS2_KYO1	-72.18	-40.65	Shoot	10283	209289	49.13	Forest	10.2	1403	NA	Chile	*Amomyrtus luma*
Alternaria avenicola	e6c1a3819c358547e43bf6e62ae0a52c	ITS2	6552	gITS7/ITS4	25.2214	60.7475	Shoot	4	148907	0.026862404	Forest	4.8	621	NA	Finland	*Picea abies*
Alternaria avenicola	3c8b5a02fbbc687601ff0960cf2e678a	ITS2	504	ITS_S2F + ITS3 mixed 1:1/ITS4	24.6624	60.2378	Air	137	118208	1.158974012	Forest	5.5	635	NA	Finland	
Alternaria avenicola	23749aa1bcadccae908868a7c4521df1	ITS2	875	ITS3/ITS4	-121.916	52.315	Soil	4	397148	0.010071812	Forest	4.4	565	NA	Canada	*Picea engelmannii x glauca*
Alternaria avenicola	5236f208fd10be5d961b1608716e0457	ITS2	504	ITS_S2F + ITS3 mixed 1:1/ITS4	24.6624	60.2378	Air	137	118208	1.158974012	Forest	5.5	635	NA	Finland	

Alternaria avenicola	ff7d6ca58c83d23c17241f382a4234cf	ITS2	504	ITS_S2F + ITS3 mixed 1:1/ITS4	24.6624	60.2378	Air	137	118208	1.158974012	Forest	5.5	635	NA	Finland	
Alternaria avenicola	a5a850653b6a9d5be42d69160182aba4	ITS2	7300	ITS_S2F + ITS3 mixed 1:1/ITS4	29.893	62.6343	Air	35	52210	0.670369661	Anthropogenic	3.3	594	NA	Finland	
Alternaria avenicola	2db75a55c5b1c1171c2d2e400c626d91	ITS2	4370	ITS_S2F + ITS3 mixed 1:1/ITS4	30.1733	62.6131	Air	47	92082	0.51041463	Forest	2.5	731	NA	Finland	
Alternaria avenicola	c6a251dfd5659973dc196dfc0b9a80a2	ITS2	358	ITS_S2F + ITS3 mixed 1:1/ITS4	23.7617	61.4997	Air	127	144059	0.88158324	Anthropogenic	4.4	619	NA	Finland	
Neostemphylium polymorphum	1d0cffb55ac621d9de77f5c45600d6ba	ITS2	4773	ITS1F/ITS4	147.614	-42.279	Soil	2	156717	0.0127	Grassland	10.9	615	5.5	Australia	
Neostemphylium polymorphum	58042f86d5ec0f8c17915130192fa283	ITS2	16963	5.8S_Fun/ITS4_Fun	2.95	45.77	Air	2	22821	0.087	Grassland	6.9	1214	NA	France	
Neostemphylium polymorphum	66b6a8c0cadfbd99ada97bdb3b24678b	ITS2	4773	ITS1F/ITS4	147.614	-42.279	Soil	1	156717	0.0063	Grassland	10.9	615	5.5	Australia	
Neostemphylium polymorphum	87e8163b46c22ddb7d63dddc9aa3ec31	ITS2	4773	ITS1F/ITS4	147.614	-42.279	Soil	29	156717	0.1850	Grassland	10.9	615	5.5	Australia	
Neostemphylium polymorphum	967cf79dc9ad99998975be9c8c882b60	ITS2	4773	ITS1F/ITS4	147.614	-42.279	Soil	35	156717	0.2233	Grassland	10.9	615	5.5	Australia	
Neostemphylium polymorphum	aa0d5ccd7a43d4783d0f728ca1a357e1	ITS2	16963	5.8S_Fun/ITS4_Fun	2.95	45.77	Air	4	22821	0.1752	Grassland	6.9	1214	NA	France	
Neostemphylium polymorphum	b0447c80056dc125a72c960af9a17a63	ITS2	4773	ITS1F/ITS4	147.614	-42.279	Soil	1	156717	0.0063	Grassland	10.9	615	5.5	Australia	
Neostemphylium polymorphum	cad286512c25efa342c6474fc4db7956	ITS2	4773	ITS1F/ITS4	147.614	-42.279	Soil	29	156717	0.1850	Grassland	10.9	615	5.5	Australia	
Pyrenophora seminiperda	34d8bd9bdf3662372c839eb02d204be8	ITS2	2846	Fits7/ITS4	-112.552	37.1134	Soil	648	98745	6.5623	Shrubland	12.3	265	8.44	USA	
Pyrenophora seminiperda	4f65387693d7a71a603a1f3c8f908f35	ITS2	2924	ITS86F/ITS4	-2.97938	37.081	Rhizosphere soil	898	165454	5.42749042	Shrubland	5.9	551	5.83	Spain	*Thymus zygis*
Pyrenophora seminiperda	0a1137944cec67aa407959730f6a3ae5	ITS2	20849	ITS7o/ITS4	-112.523	36.625	Root	79	19916	3.966659972	Shrubland	11.9	349	NA	USA	*Achnatherum hymenoides*
Pyrenophora seminiperda	6330ed88ce72d7988d5e0eab985ec917	ITS2	4839	ITS86F/ITS4	-29.7962	37.0816	Soil	285	126643	2.250420473	Shrubland	5.9	551	6.21	Spain	*Thymus zygis*
Pyrenophora seminiperda	4dae324039de11d95d6b5517fba893c9	ITS2	2924	ITS86F/ITS4	-29.7938	37.081	Rhizosphere soil	898	165454	5.42749042	Shrubland	5.9	551	5.83	Spain	*Thymus zygis*
Pyrenophora seminiperda	34d8bd9bdf3662372c839eb02d204be8	ITS2	2846	fITS7/ITS4	-112.552	37.1134	Soil	648	98745	6.562357588	Shrubland	12.3	265	8.44	USA	
Pyrenophora seminiperda	b1a64842f68814bee91d97dd1e65f4be	ITS2	12135	ITS7o/ITS4	-112.523	36.625	Rhizosphere soil	68	25765	2.639239278	Shrubland	11.9	349	NA	USA	*Bromus tectorum*

Pyrenophora seminiperda	a81ce44ad32be41fb9a5c8c5fd5a8eec	ITS2	14958	ITS7o/ITS4	-112.523	36.625	Root	32	28169	1.136000568	Shrubland	11.9	349	NA	USA	*Bromus tectorum*
Pyrenophora seminiperda	5604ab565654129169f144c39537f0f6	ITS2	2924	ITS86F/ITS4	-2.97938	37.081	Rhizosphere soil	898	165454	5.42749042	Shrubland	5.9	551	5.83	Spain	*Thymus zygis*
Pyrenophora seminiperda	502bd02790472cee57bb4b47d586c3a4	ITS2	2924	ITS86F/ITS4	-2.97938	37.081	Rhizosphere soil	898	165454	5.42749042	Shrubland	5.9	551	5.83	Spain	*Thymus zygis*
Scleromyces submersus	2d973fbe9f0d95ebbc81d928215738d4	ITS2	3019	ITS1/ITS4	138.727	-34.933	Soil	20	129610	0.1543	Woodland	13.5	938	5.4	Australia	
Scleromyces sp. ITS2-ENV1	05131d3b383aaa1ba73a69bc6a7ffdf4	ITS2	8507	ITS9/ITS4	-121.639	39.4532	Root	2	193875	0.0103	Aquatic	18	702	NA	USA	*Lilaea scilloides*
Scleromyces sp. ITS2-ENV1	1f21676d35ceed7c84e8efbc10653dc6	ITS2	12244	ITS9/ITS4	-121.629	39.5153	Shoot	1	7333	0.1363	Aquatic	17.9	799	NA	USA	*Najas guadalupensis*
Scleromyces sp. ITS2-ENV1	2f7eb730070aaa21b9567fea4badb5fb	ITS2	8507	ITS9/ITS4	-121.639	39.4532	Root	2	193875	0.0103	Aquatic	18	702	NA	USA	*Lilaea scilloides*
Scleromyces sp. ITS2-ENV1	4b24f86b45d28909fb24ff9233ba6498	ITS2	8507	ITS9/ITS4	-121.639	39.4532	Root	2	193875	0.0103	Aquatic	18	702	NA	USA	*Lilaea scilloides*
Scleromyces sp. ITS2-ENV1	597ac7d83b3c6f1a5f35918e021f1366	ITS2	18027	ITS9/ITS4	-121.629	39.5153	Shoot	22	18189	1.2095	Aquatic	17.9	799	NA	USA	*Potamogeton*
Scleromyces sp. ITS2-ENV1	6e25c9dddb6bccc05a7f858a6b5db5ae	ITS2	4446	ITS9/ITS4	-121.629	39.5153	Shoot	2	6324	0.3162	Aquatic	17.9	799	NA	USA	*Potamogeton*
Scleromyces sp. ITS2-ENV1	79ed1e9a54df669435f5285e7f9a8ae5	ITS2	8507	ITS9/ITS4	-121.639	39.4532	Root	2	193875	0.0103	Aquatic	18	702	NA	USA	*Lilaea scilloides*
Scleromyces sp. ITS2-ENV1	8fd4e6fb95f61cf85968364bbf094eeb	ITS2	8507	ITS9/ITS4	-121.639	39.4532	Root	2	193875	0.0103	Aquatic	18	702	NA	USA	*Lilaea scilloides*
Scleromyces sp. ITS2-ENV1	b24de2e10386ebb855cd45fdf54547bb	ITS2	4446	ITS9/ITS4	-121.629	39.5153	Shoot	1	6324	0.1581	Aquatic	17.9	799	NA	USA	*Potamogeton*
Scleromyces sp. ITS2-ENV1	c3698cd312b026411f3b96f4e25116c5	ITS2	4446	ITS9/ITS4	-121.629	39.5153	Shoot	1	6324	0.1581	Aquatic	17.9	799	NA	USA	*Potamogeton*
Scleromyces sp. ITS2-ENV1	c99809a0b12c3b35ea5f8ec085c30a9b	ITS2	8507	ITS9/ITS4	-121.639	39.4532	Root	2	193875	0.0103	Aquatic	18	702	NA	USA	*Lilaea scilloides*
Scleromyces sp. ITS2-ENV1	c9da1c75383e07ae0f29cb7ebd34d6ec	ITS2	8507	ITS9/ITS4	-121.639	39.4532	Root	2	193875	0.0103	Aquatic	18	702	NA	USA	*Lilaea scilloides*
Scleromyces sp. ITS2-ENV1	d6383b786a2c973a11a4cdc9d5b1ae20	ITS2	18027	ITS9/ITS4	-121.629	39.5153	Shoot	2	18189	0.1099	Aquatic	17.9	799	NA	USA	*Potamogeton*
Scleromyces sp. ITS2-ENV1	f55ed07fed4032f35404f6db08d712de	ITS2	8507	ITS9/ITS4	-121.639	39.4532	Root	2	193875	0.0103	Aquatic	18	702	NA	USA	*Lilaea scilloides*
Scleromyces sp. ITS2-ENV1	ff741397fc8be27effa06612142e6d21	ITS2	8507	ITS9/ITS4	-121.639	39.4532	Root	3	193875	0.0154	Aquatic	18	702	NA	USA	*Lilaea scilloides*
Stemphylium lycopersici	5b0276f3fffc98d811ce70855844960f	ITS2	16825	ITS3ngs10/ITS4ngs	-5.66	-15.94	Soil	75	41513	1.806	Woodland	19.3	888	5.13	UK. St. Helena	*Commidendrum robustum*

Stemphylium lycopersici	6120a99178a8b20be667f161838732be	ITS2	6378	fITS7/ITS4	114.65	22.57	Shoot	208	106412	1.9546	Shrubland	23.2	1929	NA	China	*Mussaenda kwangtungensis*
Stemphylium lycopersici	bac41123745959ccb38a31220ca5d676	ITS2	14779	ITS3ngs10/ITS4ngs	-5.66	-15.94	Soil	9	33323	0.2700	Woodland	19.3	888	5.58	UK. St. Helena	*Commidendrum robustum*
Stemphylium lycopersici	cd4d40a0ef355f976209101ee0379a70	ITS2	6384	fITS7/ITS4	114.65	22.57	Shoot	47	101043	0.4651	Shrubland	23.2	1929	NA	China	*Mussaenda kwangtungensis*
Stemphylium lycopersici	fcc7eac996d571f7843d9edd7ad60ca3	ITS2	19864	ITS3ngs10/ITS4ngs	-5.66	-15.946	Soil	129	18609	6.9321	Woodlad	19.3	888	4.26	UK. St. Helena	*Commidendrum robustum*
Stemphylium vesicarium	0f7ad62712c422213b54f8eb22b16bf0	ITS2	2866	ITS2F/ITS2R	-71.13	-34.37	Shoot	4	80863	0.0494	Cropland	16.4	507	NA	Chile	*Vitis vinifera*
Stemphylium vesicarium	1f40a73ffcd46ae42a78ca91b1eef985	ITS2	21716	ITS2F/ITS2R	-71.4	-34.5	Shoot	5	27753	0.1801	Cropland	16.2	640	NA	Chile	*Vitis vinifera*
Stemphylium vesicarium	2c37ecf51d498309408e001b3faf688e	ITS2	1599	Fits9its4	19.3714	42.565	Root and Rhizosphere soil	2	40096	0.4988	Grassland	11.7	1839	8.06	Montenegro	*Lactuca serriola*
Stemphylium vesicarium	3237eb929429f4c1f1e9b7d71419eba1	ITS2	4363	ITS2F/ITS2R	-71.4	-34.5	Shoot	10	63473	0.1575	Cropland	16.2	640	NA	Chile	*Vitis vinifera*
Stemphylium vesicarium	383cea421e6f763f3015359d40e11862	ITS2	21716	ITS2F/ITS2R	-71.4	-34.5	Shoot	3	27753	0.1080	Cropland	16.2	640	NA	Chile	*Vitis vinifera*
Stemphylium vesicarium	4687f1ae920a25ad43e08de3939eb73d	ITS2	4363	ITS2F/ITS2R	-71.4	-34.5	Shoot	18	63473	0.2835	Cropland	16.2	640	NA	Chile	*Vitis vinifera*
Stemphylium vesicarium	4e36b68177bc8ad38d07e558e5b04763	ITS2	14035	ITS2/ITS2R	-71.4	-34.5	Shoot	6	34777	0.1725	Cropland	16.2	640	NA	Chile	*Vitis vinifera*
Stemphylium vesicarium	4f4deae930db659a8aed2d42b777a0b2	ITS2	13551	ITS2/ITS2R	-71.16	-34.36	Shoot	10	72254	0.1384	Cropland	15.2	681	NA	Chile	*Vitis vinifera*
Stemphylium vesicarium	54ed190e8d9c1a7b493a136d48663954	ITS2	7953	ITS2/ITS2R	-71.13	-34.37	Shoot	21	60405	0.3476	Cropland	16.4	507	NA	Chile	*Vitis vinifera*
Stemphylium vesicarium	6c358721ebb80bc49a4c163ded84dbbc	ITS2	4358	ITS2F/ITS2R	-71.16	-34.36	Shoot	2	45733	0.0437	Cropland	15.2	681	NA	Chile	*Vitis vinifera*
Stemphylium vesicarium	7ba789aead8ae81a73ee00cf27dbe5a6	ITS2	4374	ITS86F/ITS4	-2.98623	37.0064	Rhizosphere soil	1	182418	0.0054	Shrubland	13.2	446	8.6	Spain	*Thymus zygis*
Stemphylium vesicarium	7d0b87c07c3622366e63b935c9987fc2	ITS2	1459	ITS86F/ITS4	-3.35489	36.9241	Soil	1	89525	0.0111	Shrubland	13.7	498	7.12	Spain	*Thymus zygis*
Stemphylium vesicarium	7d9f6d9f6eeba00f7497716f5fe67b9f	ITS2	6513	ITS1/ITS4	18.7537	-33.960	Shoot	7	91158	0.0767	Cropland	16.6	1041	3.61	South Africa	*Vitis vinifera*
Stemphylium vesicarium	8036cd356fad11976d311b25f9dff98e	ITS2	4378	ITS2F/ITS2R	-71.4	-34.5	Shoot	3	68788	0.0436	Cropland	16.2	640	NA	Chile	*Vitis vinifera*
Stemphylium vesicarium	84a599ffc5bcf99f59c6fa80e6c8f7ea	ITS2	7380	ITS2F/ITS2R	-71.4	-34.5	Shoot	9	155358	0.0579	Cropland	16.2	640	NA	Chile	*Vitis vinifera*
Stemphylium vesicarium	c519b0dfffcb76d8324b965099400755	ITS2	7483	ITS2F/ITS2R	-71.3	-34.37	Shoot	3	32624	0.0919	Cropland	16.4	507	NA	Chile	*Vitis vinifera*

Stemphylium vesicarium	c9daedc52d6ac1f8373d8b884 2a8a79c	ITS2	3285	ITS2F/ITS2R	-71.29	-34.3	Shoot	3	114588	0.0261	Cropland	16.3	516	NA	Chile	*Vitis vinifera*
Stemphylium vesicarium	e0bc266a227f65bff4d369b402 0421b7	ITS2	4291	ITS1/ITS4	-77.0919	43.1250	Rhizosph ere soil	12	220187	0.0454	Cropland	9.1	917	6.8	USA	*Malus domestica*

[1]Internal Transcribed Spacer region; [2] Number assigned to a particular environmental sample; [3]Abundance of a singular sequence in a particular environmental sample; [4]Total abundance of ITS sequences found in a particular environmental sample; [5]Relative abundance of a singular ITS sequence in a particular environmental sample; [6]Mean Annual Temperature (ºC); [7]Mean Annual Precipitation (mm).
